# Recurrent Acute Upper Gastrointestinal Bleed Due to Diffuse B-cell Lymphoma of the Duodenum in a Renal Transplant Patient: A Case Report and Literature Review

**DOI:** 10.7759/cureus.54555

**Published:** 2024-02-20

**Authors:** Michael Ladna, John George, Christopher E Forsmark

**Affiliations:** 1 Internal Medicine, University of Florida College of Medicine, Gainesville, USA; 2 Gastroenterology, University of Florida College of Medicine, Gainesville, USA

**Keywords:** post-transplant malignancy, diverticulitis, posttransplant lymphoproliferative disorders, upper gi bleed, b-cell lymphoma

## Abstract

A patient status post (s/p) renal transplantation in 2014 presented with an upper gastrointestinal bleed (UGIB). The source of the bleed was found to be a large mass in the duodenum with histopathology from biopsies obtained during esophagogastroduodenoscopy revealing diffuse large B-cell lymphoma (DLBCL) of the duodenum. His mycophenolate was stopped, and the tacrolimus dose was reduced due to active malignancy. He was discharged and completed one cycle of R-CHOP (rituximab, cyclophosphamide, doxorubicin, vincristine, and prednisone) before presenting back to ED with hemorrhagic shock from a large upper GI bleed requiring admission to the medical intensive care unit. Post-transplant lymphoproliferative disorders such as DBLCL can present 10 years from the transplant date. These malignancies are at high risk for bleed, especially after treatment with chemotherapy is initiated.

## Introduction

Renal transplant patients have a higher risk of malignancy and infection due to long-term immunosuppression [1]. The second most common cause of death in solid organ transplant patients is malignancy right after cardiovascular disease [2]. This patient population is at especially high risk of developing post-transplant lymphoproliferative disorders (PTLDs) such as diffuse large B-cell lymphomas (DLBCLs). DLBCLs of the gastrointestinal (GI) tract are rare malignancies, especially in the duodenum [3]. The presence of these malignancies in the GI tract increases the risk of a GI bleed, perforation, and stricture, especially after exposure to systemic chemotherapy and radiation [4]. The median time to development is 133 months or approximately 11 years [2]. Thus, even when a patient is a decade or longer after transplantation, clinicians should still be aware of the high risk of developing PTLD such as DLBCL. In addition, these patients must be closely followed and monitored once chemotherapy is initiated due to the high risk of adverse events in the form of bleed or perforation.

## Case presentation

**Figure 1:**
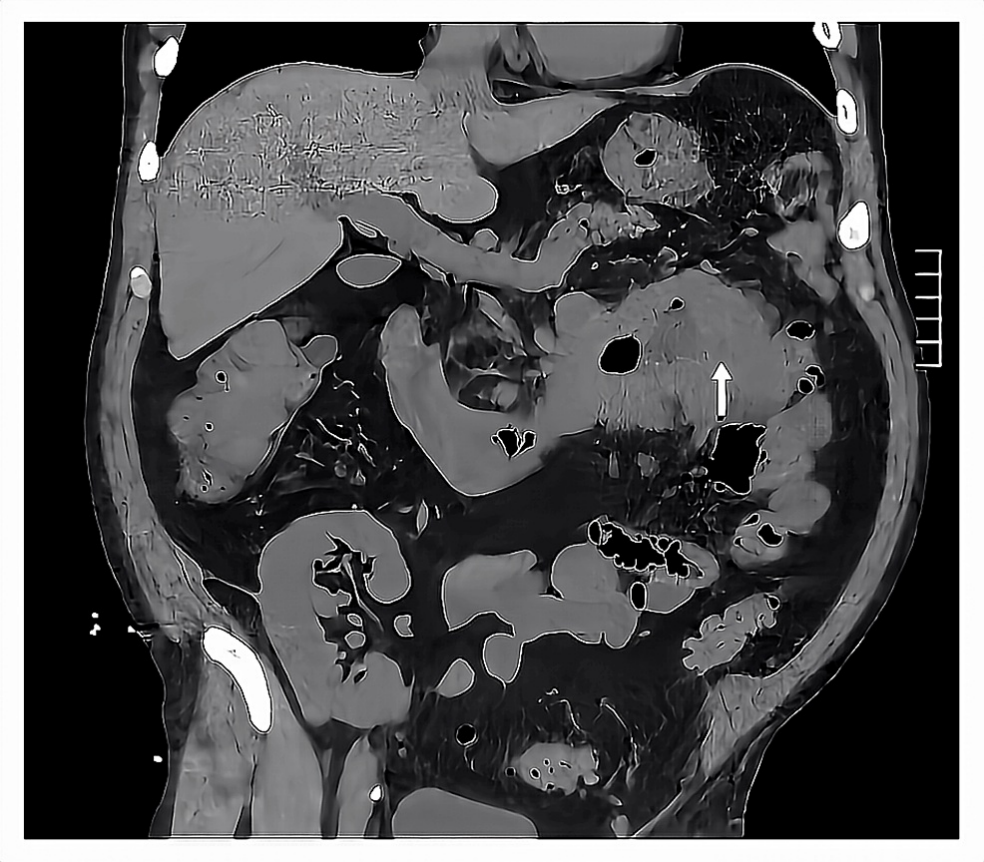
CT of the abdomen and pelvis showing a large mass in the duodenum (white arrow).

A male in his 50s with a past medical history of parathyroid cancer status post (s/p) resection and radiation therapy resulting in acquired hypothyroidism, end-stage renal disease (ESRD) due to chronic nephrosclerosis s/p living-related kidney transplantation (LRKT) in 2014, hypertension, hyperlipidemia, and gastroesophageal reflux disease presented to the emergency department (ED) on the instruction from a provider at an urgent care clinic where he was found to be hypotensive and in sinus tachycardia to 150s. He reported two months of black tarry stools, followed by two weeks of intermittent symptomatic hypotension as low as 80/60 mmHg associated with presyncope without syncope and dyspnea on exertion. He then developed watery diarrhea for three days, which led to an urgent care clinic appointment. In the ED, he was diagnosed with symptomatic acute blood loss anemia with initial hemoglobin (Hgb) of 6.7 g/dl requiring one unit of packed red blood cells (pRBC) and sepsis requiring initiation of broad-spectrum antibiotics with cefepime, metronidazole, and vancomycin. His blood pressure and heart rate normalized after blood products and aggressive fluid resuscitation. The patient left against medical advice and was informed over the telephone one day later that the blood cultures were growing gram-negative rods (GNR) and that he should immediately present back to the ED. He returned to the ED and was found to be septic once again. He was given 30 mL/kg bolus of isotonic fluid and was started on broad-spectrum IV antibiotics with cefepime, metronidazole, and vancomycin. He reported the persistence of melena. A complete blood count (CBC) from the second ED presentation revealed a Hgb of 7.6 g/dl and a basic metabolic panel (BMP) showed a stable creatinine of 0.9 mg/dl without evidence of acute kidney injury or transplant rejection. Computed tomography (CT) of the abdomen and pelvis showed acute uncomplicated sigmoid diverticulitis as well as a large mass in the duodenum, concern for peritoneal carcinomatosis, regional lymphadenopathy, and likely metastasis to the liver and lungs (Figure 1).

**Figure 2:**
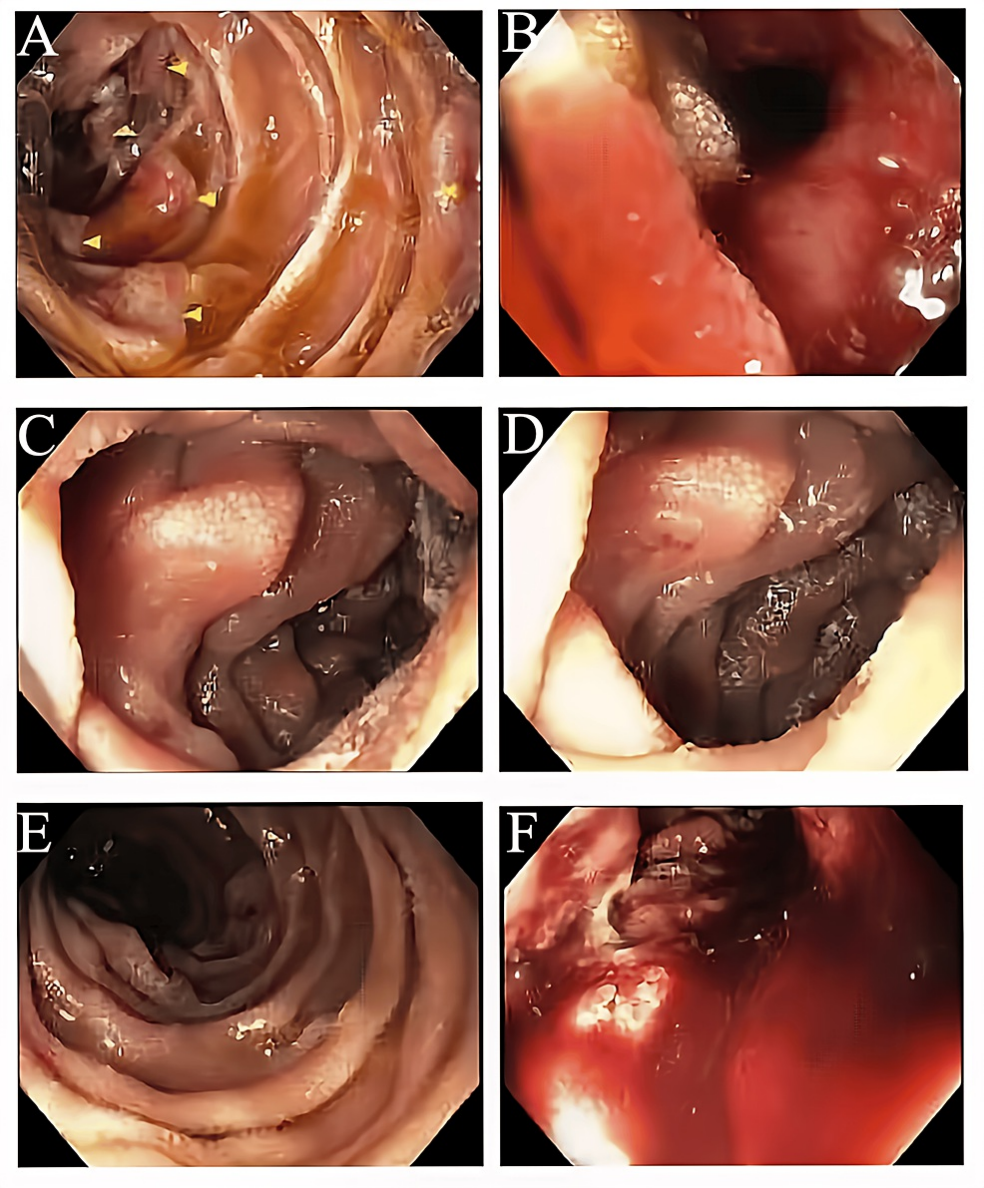
Upper endoscopy showing a large ulcerated mass (A, C, D, E) with active bleeding in the third portion of the duodenum (B, F).

This was his first bout of acute diverticulitis. He was started on an intravenous (IV) proton pump inhibitor (PPI) twice daily for the management of the upper GI bleed. Gastroenterology was consulted and performed an esophagogastroduodenoscopy (EGD) on day two of hospitalization, which showed a large ulcerated mass with active bleeding in the third portion of the duodenum for which several hemostatic sprays were applied to achieve hemostasis (Figure 2). There were also two 10-20 mm nodules with no bleeding or stigmata of recent bleeding in the gastric body and two 20 mm flat oval lesions in the duodenal bulb. Multiple biopsies of nodules in the gastric body, oval lesions in the duodenal bulb, and large ulcerated mass were taken.

Histopathology of all biopsy specimens showed aggressive B-cell lymphoma of germinal center origin. GI polymerase chain reaction (PCR) of stool detected campylobacter. *Clostridium difficile* toxin PCR was negative. Initial blood cultures from the first ED visit were speciated as *Eikenella corrodens*; however, repeat blood cultures from the second ED visit did not yield any growth. A transthoracic echocardiogram (TTE) was done to evaluate for infective endocarditis but did not show any valvular vegetation. Vancomycin was stopped due to a lack of evidence of methicillin-resistant *Staphylococcus aureus* (MRSA) infection. Transplant infectious disease (ID) was consulted and recommended discontinuing cefepime and metronidazole and initiation of ampicillin-sulbactam for the remainder of hospitalization, followed by oral amoxicillin-clavulanate for a total of two weeks of antibiotic therapy for the acute uncomplicated sigmoid diverticulitis. Transplant nephrology was consulted and due to the active malignancy and infection recommended discontinuation of the mycophenolate, and a reduction in the dose and target FK506 level (decreased to 3-5 ng/mL from 5-7 ng/mL) of the tacrolimus. Of note, he denied a history of supratherapeutic FK506 levels. The patient was discharged home after a 13-day hospitalization.

He followed up with an outside oncologist and completed one cycle of R-CHOP (rituximab, cyclophosphamide, doxorubicin, vincristine, and prednisone). Shortly after completion of the first cycle of R-CHOP and approximately one month after discharge, he was re-admitted to an outside hospital for hemorrhagic shock from a recurrent upper GI bleed originating from the duodenal lymphoma. He was sedated, intubated, and placed on mechanical ventilation. Massive transfusion protocol was initiated and he required a total of nine units of pRBCs, four units of platelets, and four units of fresh frozen plasma (FFP). Both norepinephrine and vasopressin were needed to maintain mean arterial pressure > 65. He then underwent an EGD with hemostatic sprays applied to the oozing circumferential bleed at the site of duodenal lymphoma, followed by a gastroduodenal artery embolization via the celiac artery with interventional radiology (IR). Despite those interventions, his Hgb continued to trend downward and thus he was transferred to our academic hospital. Once transferred, IR, acute care surgery, and medical oncology were consulted; however, the patient was deemed to not be a candidate for further invasive interventions aside from chemotherapy and endoscopy. He was transferred back to the outside hospital to resume care and optimization for chemotherapy.

## Discussion

GI lymphoma is a relatively rare tumor accounting for only 1-8% of malignant neoplasms of the GI tract [5,6]*.* Primary GI lymphomas comprise 10-15% of all non-Hodgkin lymphomas (NHLs) and encompass 30-40% of all total extranodal lymphomas [7]. The majority of GI lymphomas occur in the stomach, making up approximately 60-75% of cases, with duodenal involvement being rare [7]. The prevalence of primary gastric, small intestinal, and duodenal lymphomas is 10:3:1 [3]. Retrospective analysis of 126 patients with intestinal DLBCL revealed that the ileocecal region was the most commonly involved (50.0%), followed by the jejunum or ileum (23.0%), duodenum (18.3%), colon (11.1%), and finally rectum (5.6%) [8]*.* The most common duodenal lymphoma is follicular lymphoma accounting for 41.1% of cases followed by DLBCL accounting for 32.8% of cases [9]. Approximately 30% of duodenal DLBCLs were associated with gastric lesions, which was the case in our patient [8]. The typical morphology of duodenal DLBCL visualized on EGD is classically an ulcerative and protruding lesion [10]. The neoplastic cells of DLBCL tend to be found throughout the entire thickness of the GI tract, which increases the risk of intestinal perforation, bleeding, and stricture, especially after the initiation of chemotherapy [4]*.*

The second most common cause of death in renal transplant patients after cardiovascular disease is malignancy with projections, indicating it may become the most common cause of death within the next two decades [2]. One study found that the incidence of cancer in renal transplant recipients was 4% [2]. Cancer-related mortality is higher in solid organ transplant patients compared to the general population [11]. Compared to other malignancies, PTLDs had the longest median time to development at 133 months after transplantation and the best graft survival rates [2]. The increased risk of malignancy in renal transplant patients is largely attributed to the effects of immunosuppression, which impairs T-cell function and decreases immunologic control of oncologic viral infections such as Epstein-Barr virus (EBV) [1]. PTLD tends to occur in a bimodal distribution after transplantation, suggesting an early-onset and late-onset version. Risk factors for early-onset PTLD were younger age at transplantation (HR: 3.97), as well as recipient seronegative status for EBV (HR: 3.13) and cytomegalovirus (CMV) (HR: 1.49). Recipient seronegative status for EBV and CMV were not associated with an increased risk for late-onset PTLD [12]. Treatment of renal transplant rejection further hinders T-cell function and has been linked to an increased risk of PTLD with exposure to anti-lymphocyte antibodies being associated with an increased risk of NHL [13]. A retrospective study of 366 renal transplant patients from Japan identified treatment with tacrolimus (HR: 4.376) as a risk factor for malignancy [14]. In liver transplant patients, higher serum tacrolimus levels were associated with lower PTLD survival [15]. It is unknown whether higher tacrolimus levels are associated with a higher risk of developing PTLD than lower tacrolimus levels. More than 50% of PTLD cases are EBV-related and a mismatch between the donor and recipient (EBV-negative receptor engrafted with an EBV-positive donor) is associated with a 35% and 42% increase in PTLD incidence in deceased-donor and living-donor kidney transplantations, respectively [16].

DLBCL is the most common form of NHL accounting for 30-40% of all NHL [17]. Our patient presented with advanced disease, which is unfortunately the case for the majority of patients since 70% of all DLBCL are first diagnosed as advanced disease. The standard of care for the treatment of advanced DLBCL is six cycles of R-CHOP administered every three weeks, which has a 60-70% cure rate [17]. Recently, POLA-R-CHP (polatuzumab, vedotin, rituximab, cyclophosphamide, doxorubicin, and prednisone) has emerged as an alternative regimen in light of recent data from the phase 3 POLARIX trial, which showed superior two-year progression-free survival of 74.2% versus 66.5% with POLA-R-CHP and R-CHOP, respectively [18]. Radiation therapy is not typically used and reserved for a subset of patients who already received standard-of-care treatment, do not have disease progression but do have residual positive sites on positron emission tomography (PET) scans. Of the patients, 20-25% are not candidates for first-line treatment with R-CHOP, typically due to poor fitness, coexisting medical conditions, or significant cardiac dysfunction. For these patients, a reduced-dose version of R-CHOP, also known as R-mini CHOP, can be considered. A short pretreatment course with glucocorticoids, with or without vincristine, can be given to improve the adverse effect profile associated with R-CHOP. If there is a contraindication to anthracycline, substitution with gemcitabine or etoposide can be made [19].

There is limited data on the treatment of DLBCL PTLD in renal transplant patients. The remission rate was 76% for DLBLC PTLD in all solid organ transplant patients treated with R-CHOP. Treatment-related mortality was 7% and all deaths were attributed to sepsis. Graft rejection was suspected in eight (9%) patients with four (4%) being confirmed on biopsy and no cases of graft loss. Only one patient had confirmed graft rejection during treatment with R-CHOP with the others occurring after completion of treatment. It was difficult to determine whether graft rejection was due to chemotherapy, dose reduction of immunosuppressants, or a combination of both [20].

## Conclusions

Our case was particularly unique in that the PTLD originated in the duodenum, which is an uncommon site for malignancies, especially lymphomas. In addition, the DLBCL was EBV negative. The EBV status of the donor was unknown since the kidney transplant was done at an outside facility and we were unable to acquire those records. This case also demonstrated the risks of chemotherapy for GI malignancies, which in our patient was recurrent upper GI bleeding that led to hemorrhagic shock. Clinicians should be aware that PTLD can present a decade or more out from the transplantation date and involvement of the GI tract increases the risk of some very serious complications in the form of GI bleed, stricture, and even perforation, especially after the initiation of treatment with systemic chemotherapy. As such, this patient population should be especially closely followed throughout their disease course.
